# Anticancer efficacy of a supramolecular complex of a 2-diethylaminoethyl–dextran–MMA graft copolymer and paclitaxel used as an artificial enzyme

**DOI:** 10.3762/bjnano.5.238

**Published:** 2014-12-01

**Authors:** Yasuhiko Onishi, Yuki Eshita, Rui-Cheng Ji, Masayasu Onishi, Takashi Kobayashi, Masaaki Mizuno, Jun Yoshida, Naoji Kubota

**Affiliations:** 1 Ryujyu Science Corporation, 39-4 Kosora-cho, Seto, Aichi 489-0842, Japan; 2 Department of Infectious Disease Control, Faculty of Medicine, Oita University, 1-1 Idaigaoka, Hasama-machi, Yufu, Oita 879-5593, Japan; 3 Department of Human Anatomy, Faculty of Medicine, Oita University, 1-1 Idaigaoka, Hasama-machi, Yufu, Oita 879-5593, Japan; 4 The Center for Advanced Medicine and Clinical Research, Nagoya University Hospital, 65 Tsurumai-cho, Showa-ku, Nagoya, Aichi 466-8560, Japan; 5 Chubu Rosai Hospital, Japan Labour Health and Welfare Organization, 1-10-6 Komei, Minato-ku, Nagoya, Aichi 455-8530, Japan; 6 Department of Chemistry, Faculty of Medicine, Oita University, 1-1 Idaigaoka, Hasama-machi, Yufu, Oita 879-5593, Japan

**Keywords:** artificial enzyme, diethylaminoethyl–dextran–MMA, graft copolymer, multi-drug resistance of cancer cells, paclitaxel, supramolecular complex

## Abstract

The anticancer efficacy of a supramolecular complex that was used as an artificial enzyme against multi-drug-resistant cancer cells was confirmed. A complex of diethylaminoethyl–dextran–methacrylic acid methylester copolymer (DDMC)/paclitaxel (PTX), obtained with PTX as the guest and DDMC as the host, formed a nanoparticle 50–300 nm in size. This complex is considered to be useful as a drug delivery system (DDS) for anticancer compounds since it formed a stable polymeric micelle in water. The resistance of B16F10 melanoma cells to PTX was shown clearly through a maximum survival curve. Conversely, the DDMC/PTX complex showed a superior anticancer efficacy and cell killing rate, as determined through a Michaelis–Menten-type equation, which may promote an allosteric supramolecular reaction to tubulin, in the same manner as an enzymatic reaction. The DDMC/PTX complex showed significantly higher anticancer activity compared to PTX alone in mouse skin in vivo. The median survival times of the saline, PTX, DDMC/PTX4 (particle size 50 nm), and DDMC/PTX5 (particle size 290 nm) groups were 120 h (treatment (T)/control (C), 1.0), 176 h (T/C, 1.46), 328 h (T/C, 2.73), and 280 h (T/C, 2.33), respectively. The supramolecular DDMC/PTX complex showed twice the effectiveness of PTX alone (*p* < 0.036). Above all, the DDMC/PTX complex is not degraded in cells and acts as an intact supramolecular assembly, which adds a new species to the range of DDS.

## Review

### Introduction

As a means of delivering a drug to a target effectively, the enhanced permeation and retention (EPR) effect and reticuloendothelial system (RES) were enabled by using a polymer drug delivery system (DDS), and it is thought to represent an outstanding drug delivery method [1–3].

Recent detailed research of Maysinger et al. measuring the intracellular distribution of fluorescently labeled polymer micelles by using confocal laser scanning microscopy has shown the effect of a drug administered with a polymer DDS. As for polymer micelles carrying a drug, they have been shown to be transported to and act on not only endosomes and lysosomes but also the Golgi body and mitochondria [4]. A block copolymer micelle can be used to deliver a hydrophobic drug as a nanocarrier with water-soluble biological affinity. Knowledge of the cellular distribution of micelles is required to enable the selective delivery of a drug to a specific target at the subcellular level [4]. By means of triple-labeling confocal microscopy of living cells, Savic et al. identified the exact cellular targets of block copolymer micelles, i.e., several cytoplasmic organelles, including mitochondria. Acting as drug carriers, these micelles affect the cellular distribution of the drug, as well as effectively increase the total quantity of the drug delivered to the cell [4]. It has been shown that by conjugating a drug to a copolymer carrier, the medicinal effects of the active agent can change significantly. In a study about the treatment of A2780 human ovarian carcinoma cells with geldanamycin (GA), 17-(3-aminopropylamino)-17-demethoxygeldanamycin (AP-GA), and a *N*-(2-hydroxypropyl)methacrylamide copolymer/AP-GA conjugate (P[AP-GA]), their differential effects were revealed by gene expression array analysis [5]. Remarkably, AP-GA-treated cells exhibited an increased expression of HSP70 and HSP27 compared to cells treated with GA and P[AP-GA] [5]. It was proposed that the conjugation of AP-GA to the *N*-(2-hydroxypropyl)methacrylamide copolymer resulted in the modulation of the AP-GA-triggered stress responses in cells because of the differences in the internalization mechanism, subcellular localization, and concentration gradients in cells [5–6].

Above all, the DDS complex must be not degraded in cells and act as an intact object. Nishiyama et al. [7] analyzed expression profiles of 807 genes of non-small cell lung cancer PC-14 cells after treatment with cisplatin (CDDP) incorporated in polymeric micelles (CDDP/m) versus free cisplatin. A total of 50 genes of significant differential expression between cells treated with free CDDP and CDDP/m were identified by principal component analysis with the unpaired *t*-test. Most notably, it was found that CDDP/m down-regulated the expression of genes encoding integrins and matrix metalloproteinases, which are intimately linked to the processes of tumor invasion, metastasis, and angiogenesis; in contrary, the free CDDP up-regulated the expression of these genes. These results demonstrated the capability of polymer carriers to modulate the treatment or introduce new therapeutic effects compared to the treatment with the free drug [7].

### Supramolecular objects

The phenomenon of such combination between a polymer DDS and a gene or drug shows the possibility that they act as a supramolecular object. The concept of a supramolecule was advocated by Lehn [8] and others, and the use of, for instance, crown ethers or cyclodextrins, as a host to form a host–guest compound by using intermolecular interactions has been shown. In addition, proteins, Langmuir–Blodgett films (a self-organizing film), and liquid crystals have been studied as supramolecular assemblies.

Moreover, the development of biomimetic polymers by using new supramolecular assemblies is expected, such as artificial enzymes with highly selective function, which are generated through specific interactions. Such supramolecular assemblies would have flexible characteristics, such as self-structural change, so that an enzyme may consist of a high-molecular-mass carrier and a low-molecular-weight active group, unlike a general catalyst. Moreover, they may have advantageous functional effects related to a structural change of their substrates and intermediary bodies.

### Enzymatic substrate reactions

Enzymatic substrate reactions in the human body are driven by allosteric modulation, for which changes in activity occur in combination with different ligands from an active center. Supramolecules have been suggested for the use as artificial enzymes [9]. The supramolecular diethylaminoethyl (DEAE)–dextran–methacrylic acid methylester (MMA) copolymer (DDMC)/paclitaxel (PTX) complex, which is expected to inhibit drug-induced resistance by allosteric modulation, was developed as a new type of anticancer drug. However, even if a patient is prescribed an anticancer agent, a cancer cell will soon change an antidrug gene, thereby increasing the power of multi-drug resistance (MDR) [10]. It can be imagined that the development of fatal MDR by a cancer cell to an anticancer agent can be prevented if the agent promotes positive allosteric modulation according to the enzyme reaction model of a 1:1 ratio of a substrate and enzyme, which is the ratio utilized in vivo.

### Gene delivery system

On the other hand, development of the gene delivery system in field of the genetic engineering serves as an important domain [11]. The copolymer that was used for graft polymerization of MMA onto DEAE–dextran formed a polymer micelle with a hydrophilic–hydrophobic micro-separated domain. The high transformation efficiency of this polymer micelle suggests that it could be used as a highly promising non-viral vector [12–23]. Indeed, it has been reported that a complex of the DEAE–dextran–MMA graft copolymer and DNA, which modified the properties of DEAE–dextran, had a transformation efficiency that was over 50-fold that of DEAE–dextran in various cells [17].

A supramolecular complex of DDMC/PTX was formed by using PTX as a guest and DDMC as host. Positive allosteric promotion and substrate selectivity was expected for this supramolecular complex. Indeed, the DDMC/PTX complex exerted a remarkable therapeutic effect on a PTX-resistant melanoma cell line, even at low concentrations [24]. It is thought that the supramolecular DDMC/PTX complex produced conformational flexibility on its structure, which did not generate MDR, but promoted allosteric modulation. The supramolecular DDMC/PTX complex also demonstrated substrate selectivity. This complex should be regarded as an artificial enzyme with substrate specificity, and it is reported to exert an anticancer effect as an artificial enzyme.

### Polymer micelle

**Characteristics of the 2-diethylaminoethyl-dextran methyl methacrylate graft copolymer:** DDMC is a copolymer that is formed through grafting MMA onto DEAE–dextran as the backbone polymer by using a tetravalent ceric salt [14]. It generates a polymer micelle that forms a microphase-separated structure with a hydrophilic domain for the DEAE–dextran part and a hydrophobic domain for the graft polymer PMMA.

**Figure 1:**
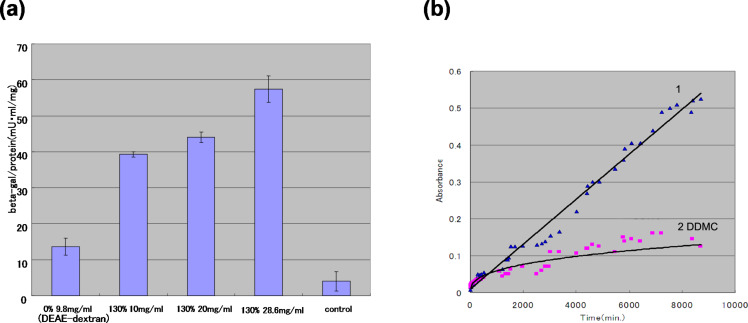
Non-viral vectoring of DEAE–dextran and DEAE–dextran–MMA graft copolymer (DDMC). (a) Transfection of DDMC into HEK293 cell lines. The grafting rate is 130% for sample 2 at 10 mg/mL, sample 3 at 20 mg/mL, and sample 4 at 28.6 mg/mL. Expression of the LacZ gene is shown at 48 h after transfection. (b) DNase degradation (1: DEAE–dextran, 2 DDMC): To the samples was added 4 mL of distilled water, then 10 u of DNase (RQ1 RNase-free DNase, Promega) and 0.1 mL of 10× reaction buffer (400 mM Tris-HCl, 100 mM MgSO_4_, 10 mM CaCl_2_, pH 8) and the samples were incubated at 37 °C. The wavelength used for this experiment was 633 nm for toluidine blue isolated from DNA. (b): Reprinted from [18].

When the transfection rate of DEAE–dextran (grafting rate 0%) and DDMC was compared by using a reporter gene (β-galactosidase) in HEK293 cells (Figure 1a), DDMC (grafting rate 130%) exhibited a remarkable increase in the transfection rate [15].

The stabilization effect of DDMC can be understood with respect to the protective effect of DEAE–dextran against the action of DNase. That is, from Figure 1b, the absorbance variation of DEAE–dextran/DNA is large, so that there is a large amount of toluidine blue because the decomposition of DNA by DNase is promoted in the presence of DEAE–dextran/DNA from the earliest stage [15]. Conversely, for DDMC/DNA, the DNA decomposition hardly progressed, but the absorbance variation was very small. It is thought that the protective action from DNase digestion is markedly larger for DDMC than for DEAE–dextran, and this resulted in the increased transfection efficiency. Therefore, it is suggested that the condensed DNA, which is taken into the cell by endocytosis, is protected from decomposition within the cell and its penetration of the nuclear membrane is also increased [11]. In addition, DDMC is considered to be more stable than DEAE–dextran to the action of the dextran-degrading enzyme dextransucrase.

The polymer micelle of DDMC forms a complex with DNA, undergoes endocytosis, and a small proportion of the complex is transported to the nucleus. However, analysis of gene expression by using DDMC/DNA has revealed that the majority of the complex is found not in the nucleus but is instead located outside of the nucleus, except during cell division, and is therefore considered to be an ideal system for transgenesis with a curative intent. Thus, it seems that it contributes to the hydrophilic–hydrophobic microphase-separated structure for the stability of DDMC. As mentioned above, when DDMC is applied as a supramolecular object for a DDS, it can be expected not to separate in the cell but to act as intact supramolecule.

### DDMC application in vivo

DDMC is very stable in suspension systems in vivo in comparison with lipofection reagents because the polymer micelle structure is stabilized at less than the critical micelle concentration. It was reported recently that the administration of DDMC as an aerosol [25] and the use of DDMC/p53 in intratumoral gene therapy [26] were effective. DDMC is very suitable for experiments using targeted siRNA or miRNA transfection in vivo. DDMC solution can be autoclaved, but autoclaving alone may not be sufficient to inactivate all RNases. DDMC solutions can be treated by adding DEPC (diethyl pyrocarbonate) to 0.05% and incubated overnight. The DDMC solutions will be removed any trace of the DEPC by autoclaving again.

## Results and Discussion

### Supramolecular DDMC/PTX complex

#### Characterization of the supramolecular DDMC/PTX complex

A complex consisting of DDMC and PTX (DDMC/PTX) was obtained by using the antitumor alkaloid PTX as the guest and DDMC as the host. The particle size distribution and ζ-potential of the DDMC/PTX complex were measured by dynamic light scattering and particle electrophoretic mobility. Furthermore, scanning electron microscopy (SEM) was used to determine the size and shape of the freeze-dried DDMC/PTX complex (DDMC/PTX5), which revealed that the complex formed uniform cubic particles with a diameter of 200–300 nm measured by dynamic light scattering. Particle size determined by SEM was 300–500 nm. The ζ-potential of the particles was +36 mV, which helps to stabilize the dispersion of the DDMC/PTX complex. To investigate the physical properties of these products, thermal and infrared analyses were also performed.

#### Thermal analysis of the supramolecular DDMC/PTX complex

**Figure 2:**
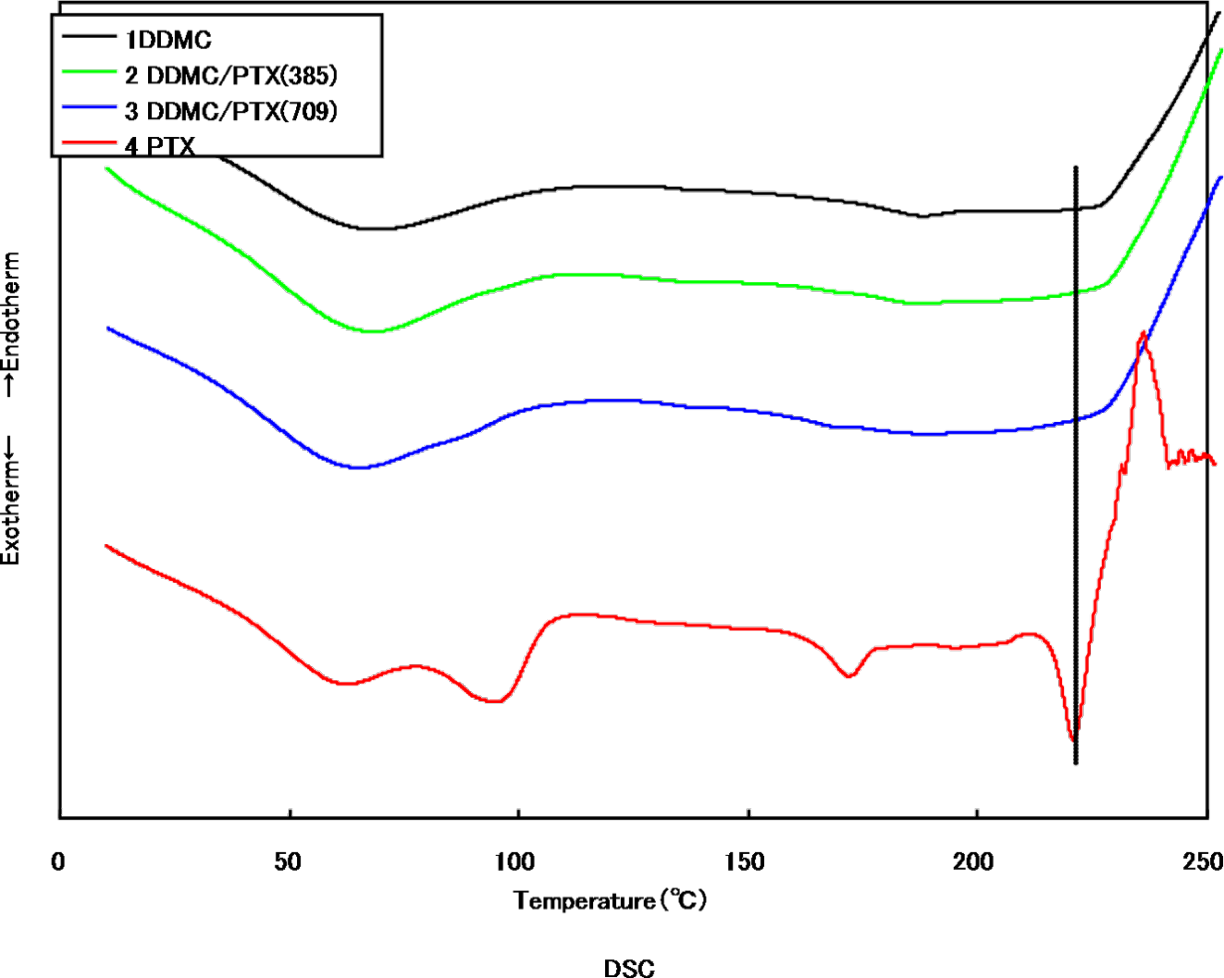
Differential scanning calorimetry (DSC) with DDMC–paclitaxel complex and paclitaxel: (1) DDMC, (2) DDMC/PTX complex (DDMC 9.6 mg/PTX 0.385 mg), (3) DDMC/PTX complex (DDMC 9.6 mg/PTX 0.709 mg), and (4) PTX. Reprinted from [62].

The differential scanning calorimetry (DSC) measurements of the DDMC/PTX supramolecular complex and PTX are shown in Figure 2.

Sample 4 exhibits three characteristic endothermic and one additional exothermic peak. A description of the peaks is given in Table 1 [27]. Conversely, in the DDMC/PTX complex samples 2 and 3, in spite of having normalized the vertical-axis scale to the PTX content, the endothermic peak at the melting point of 220.8 °C did not appear. From this, it is thought that PTX is carried as guest of the DDMC/PTX complex at the nano-level.

**Table 1:** Exothermic and endothermic peaks of PTX by DSC.^a^

*T*(I) [°C]	*T*(II) [°C]	Δ*H*(II) [mJ/mg]	*T*(III) [°C]	Δ*H*(III) [mJ/mg]	*T*(IV) [°C]

94.9	171.8	9.605	220.8	20.6056	236

^a^I: elimination of the non-structural water that is adsorbed on the sample (94.9 °C); II: dehydration of PTX hydrate (171.8 °C); III: melting point of PTX (220.8°C); Δ*H*_m_ = 20.6 mJ/mg; IV: decomposition (236 °C).

#### Infrared absorption spectrum of the supramolecular DDMC/PTX complex

**Figure 3:**
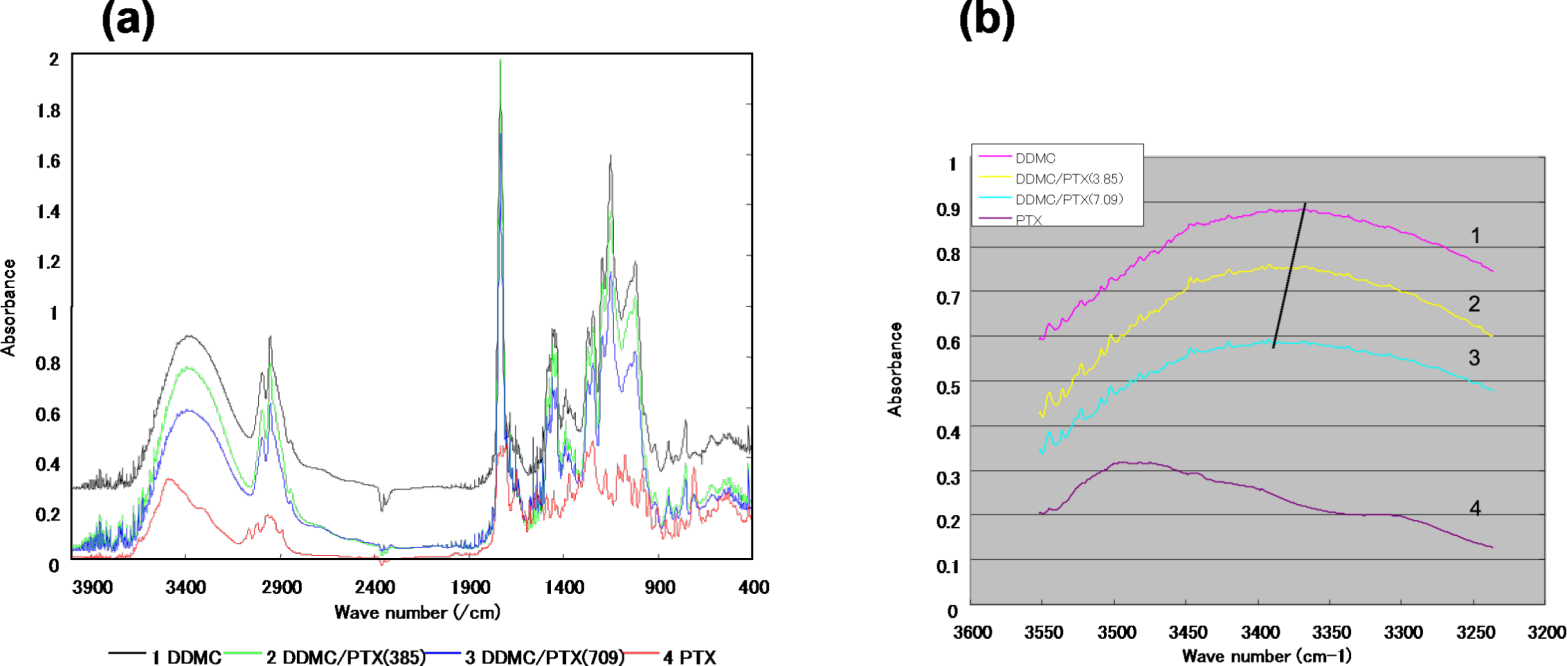
IR absorption spectra of the DDMC–paclitaxel complex and paclitaxel. (a) Mid-infrared region (4.000–400 cm^−1^), (b) X–H stretching region: (1) DDMC, (2) DDMC/PTX complex (DDMC 9.6 mg/PTX 0.385 mg), (3) DDMC/PTX complex (DDMC 9.6 mg/PTX 0.709 mg), and (4) PTX. (b): Reprinted from [62].

The infrared absorption spectra of DDMC (grafting rate: 102%), DDMC/PTX complexes, and PTX in the range of 3200–3700 cm^−1^ are shown in Figure 3a. The large and broad absorption due to the stretching vibration of N–H, O–H, and NH–O is observed at approximately 3400 cm^−1^ for the DDMC/PTX complex and DDMC, whereas it is observed in the vicinity of 3500 cm^−1^ for PTX.

Moreover, for the absorption of the disappearing crystallization water, the peaks of N–H and O–H for the DDMC/PTX complex have shifted to a higher energy than that of DDMC, the starting material. This means that the association with the hydrogen bond itself is weakened, which then reassembles the supramolecular complexes by a hydrophobic reaction between them. The escape of the crystallization water and the binding of DDMC to PTX decrease the entropy compared to the state in which it is not bound (enthalpy–entropy compensation or isokinetic theory) [28]. The enthalpy–entropy decrease is expected to stabilize the complexes against supramolecular stress, by Brownian movement owing to the law of entropy increase (effect of entropy elasticity) [29]. This is thought to be attributed to the conformational change that occurs in each supramolecular complex. However, the shift to the high energy state is remarkable for the absorption spectrum of the DDMC/PTX complex. The DDMC/PTX complex will become stable when it is folded into a three-dimensional structure by the driving force of the hydrophobic bond, and it is thought that the hydrophobic bond of the aromatic ring of PTX and the hydrocarbon backbone portion of DDMC also contribute to this process.

Figure 3b shows the more broad absorption by C–H stretching near 3000 cm^−1^ in the DDMC/PTX complex compared with DDMC. Since a shift in wavenumber will take place if an intermolecular interaction occurs, an absorption band will generally envelop the individual peaks. The hydrophobic environment of this intermolecular interaction can also be postulated from the broad absorption spectrum of the DDMC/PTX complex. From the above, Figure 3b shows the presence of a large hydrophobic bond in the DDMC/PTX complex.

#### Particle size distribution and ζ-potential

**Figure 4:**
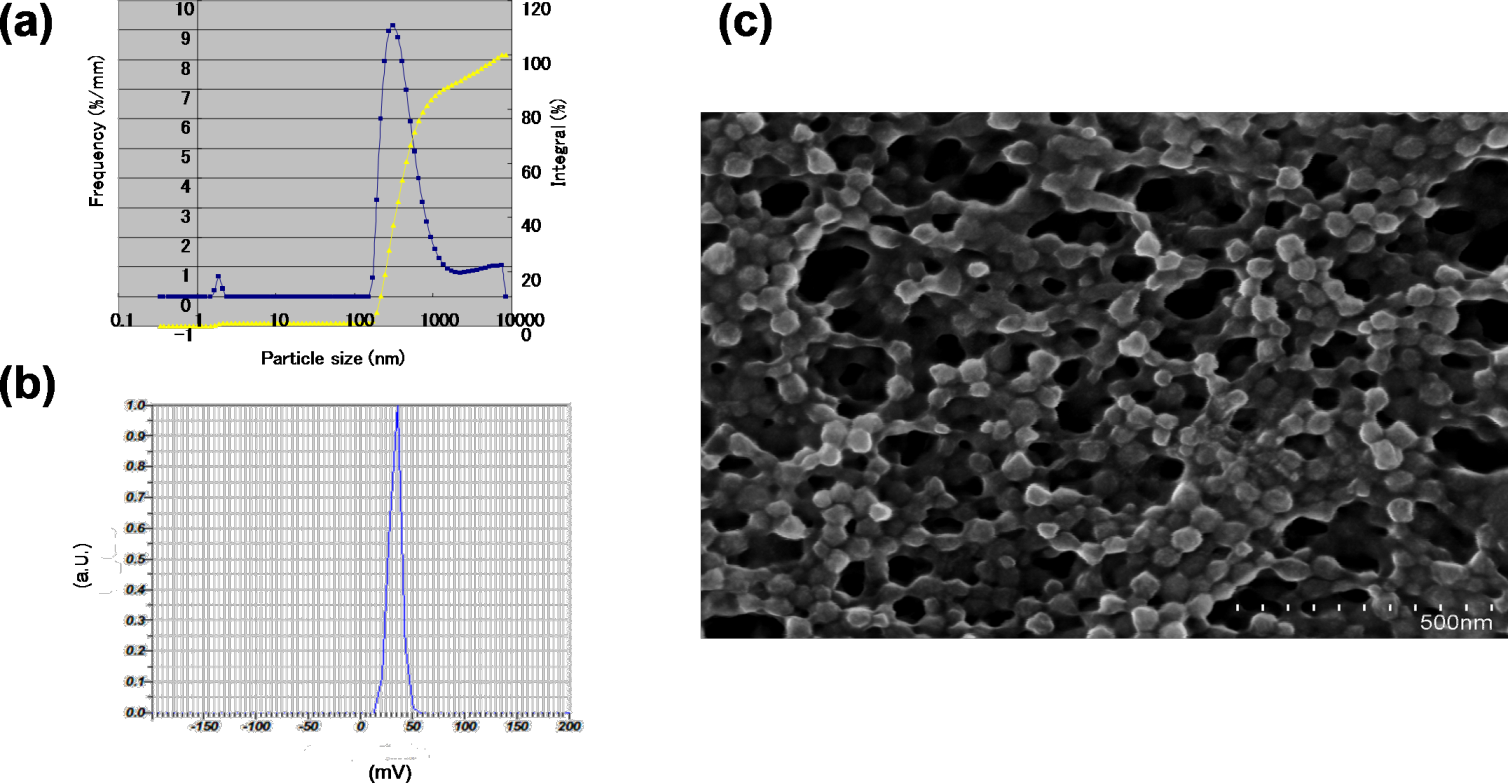
Characteristics of the DDMC–paclitaxel complex. (a) Particle size distribution and ζ-potential of the DDMC–paclitaxel complex determined by dynamic light scattering and (b) particle electrophoretic mobility measurements. (c) A Scanning electron microscopic image (HITACHI S-4800) of the freeze-dried DDMC–paclitaxel complex taken at an accelerating voltage of 5 kV. (a): Reprinted from [24].

The particle size distribution and the ζ-potential of the DDMC/PTX complex were measured by dynamic light scattering and particle electrophoretic mobility measurements (Nano Partica SZ-100). Furthermore, SEM was used to determine the size and shape of the freeze-dried DDMC/PTX complex. Dynamic light scattering revealed that the complex formed with uniform cubic particles with a diameter of 200–300 nm (Figure 4a). The particle size determined by SEM was 300–500 nm (Figure 4c), similar to that determined by dynamic light scattering. The ζ-potential of the outer layer of the particles, that is, outside of the electric double layer, was large (+36 mV; Figure 4b). However, the particle diameter of DDMC used was approximately 1200 nm because of its large association, and the ζ-potential was approximately +24 mV.

Considering that the average primary particle diameter of DDMC is approximately 25 nm, it seems that the DDMC/PTX complex forms small clusters of self-assembled supramolecular assemblies by its hydrophobic force following cleavage of the hydrogen bond. The DDMC/PTX complex, with a diameter of 200–300 nm, may make stable polymeric micelles by both the hydrophobic force and hydrogen bond. The ζ-potential of the particles, outside of the electric double layer, was +36 mV, which helps to stabilize the dispersion of the DDMC/PTX complex. Considering the high ζ-potential of the particles, it seems that the DDMC/PTX complex may have a stable hole (cave) of a hydrophobic pocket, which is required for the clathration of a substrate.

### Anticancer activity

#### Anticancer activity of the DDMC/PTX complex on melanoma B16F10 cells in vitro

In 1920, the German chemist Willstätter studied the mechanisms of enzyme reactions, and he suggested that a purified enzyme, such as saccharase, consists of a low-molecular-weight active group and a high-molecular-weight carrier [30]. The American chemist Sumner discovered, by crystallizing urease, that an enzyme is a protein and he noted that when a protein is a giant molecule, it can react as an enzyme [31]. However, different from a general catalyst, the formation of an enzyme complex is possible in the presence of large molecular subunits within the enzyme. Accordingly, biomimetic supramolecular assemblies including a guest are now defined as artificial enzymes in which the low-molecular-weight subunit containing the active site is complexed with a high-molecular-weight carrier. These supramolecular assemblies have the characteristic of conformational flexibility (slide-ring elasticity) owing to entropy elasticity [32], such as a self-structural change, so that it may become advantageous functionally according to the structural change of a substrate and an intermediary body.

#### MTT assay (WST8)

**Figure 5:**
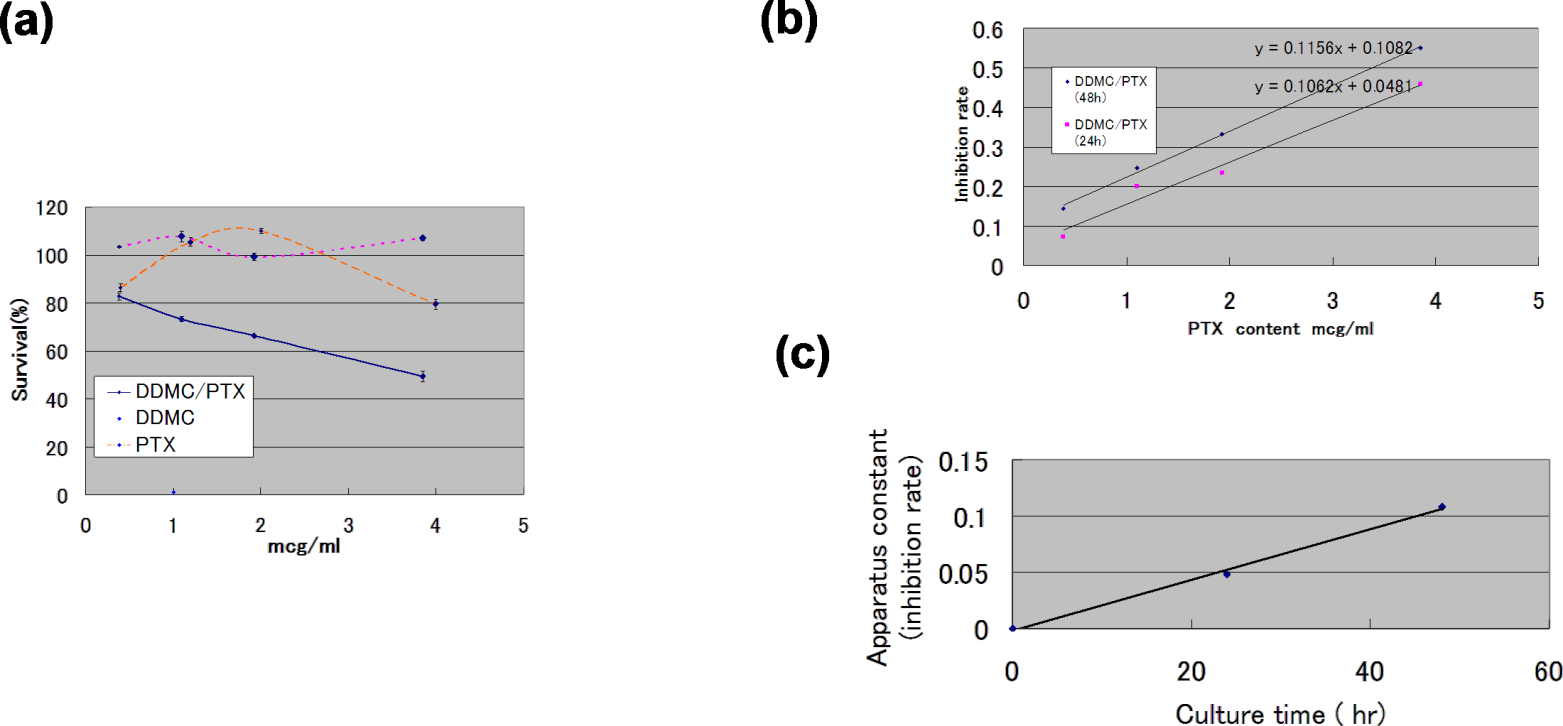
Anticancer efficacy of a supramolecular complex against PTX-resistant melanoma B16F10 cells. (a) Survival of B16F10 melanoma cells treated with paclitaxel or the DDMC–paclitaxel complex for 48 h determined by using the MTT (WST8) method (blue solid line: DDMC/PTX, purple dashed line: DDMC, orange dashed line: PTX). (b) Tubulin polymerization and cell death (Cd) rates can be expressed by enzymatic kinetic parameters. Relationship between Cd and paclitaxel concentration, [*E*]_0_, after 24 h and 48 h. After 24 h: Cd = 0.1062 [*E*]_0_ + 0.0481; after 48 h: Cd = 0.1156 [*E*]_0_ + 0.1082. (c) Apparatus constant (C_1_) by cell culture time in WST8 vs time (h) for DDMC/PTX complex. Reprinted from [24].

The WST-8 (2-(2-methoxy-4-nitrophenyl)-3-(4-nitrophenyl)-5-(2,4-disulfophenyl)-2*H*-tetrazolium) method is superior to the traditional MTT assay with regard to sensitivity and cell cytotoxicity. The results of survival analysis in vitro by a direct MTT assay (WST8) are shown for PTX-resistant melanoma B16F10 cells (Figure 5a). The resistance of this melanoma cell line to PTX changed clearly, as can be seen from the convex survival curve in Figure 5a. This convex survival curve is considered to be the product of gene expression at low concentrations of PTX (event A) and a factor negatively associated with survival at higher concentrations of PTX (event B).

It is assumed that event A is related to the MDR of the melanoma cells and event B is related to the efficacy of PTX on melanoma cells. In this way, survival probability is expressed as the product of the probability of event A (P(A)) positively and event B (P(B)) negatively, represented as P(A∩B). That is, the probability for the cells to survive increases with the probability of P(A), and decreases with P(B), with P(A) and P(B) being functions of the PTX concentration. P(A∩B) is the probability that events A and B occur in event (A,B). When events A and B are independent of each other, P(A∩B) is represented by the following formula: P(A∩B) = P(A)·P(B). For P (A∩B), the convex curve approaches P(A) and P(B) asymptotically [33].

According to Sakai [34], the MDR mechanism of cells is related to (1) the control of the intracellular uptake and extracellular discharge of a medicine; (2) changes to the metabolic system by a medicine (reduction in activation enzyme, rise of inactivation enzyme); (3) quantitative and qualitative alterations of the target molecule of the drug; (4) the microenvironment of the cancer; (5) the DNA repair system, and (6) an increase of anti-apoptosis or anti-pre-apoptosis signals. It is necessary to scrutinize these complicated factors by using gene expression analysis over time. For that purpose, “systems biology” approaches, that is, the use of system engineering analysis techniques, are needed. To understand why event A results in MDR owing to changes in gene expression by which melanoma cells survive against increasing concentrations of PTX, for example, Miyano has conducted extensive research into the resistance of these cells to PTX by using systems biology [35].

In addition, for a taxoid-based tumor-targeting drug, the resistance gene Taxol-resistant-associated protein 3 (TRAG-3) was identified in cancer cells, and it turns out that this gene is found in high amounts in solid cancers. In these studies, gene expression was measured for 24 h after treatment with PTX, which showed the presence of gene clustering over time in melanoma cells. The instructions that emerge from any gene to any gene cluster have also been analyzed in melanoma cells over time. These findings, which were obtained from dynamic Bayesian analysis in combination with non-linear regression, were confirmed by using data from DNA microarrays [36]. The survival of PTX-resistant melanoma cells has been analyzed extensively by using DNA microarrays and dynamic Bayesian analyses. A mathematical model was generated by dynamic Bayesian networks and nonparametric regression analysis using a supercomputer with 1024 cores.

**Figure 6:**
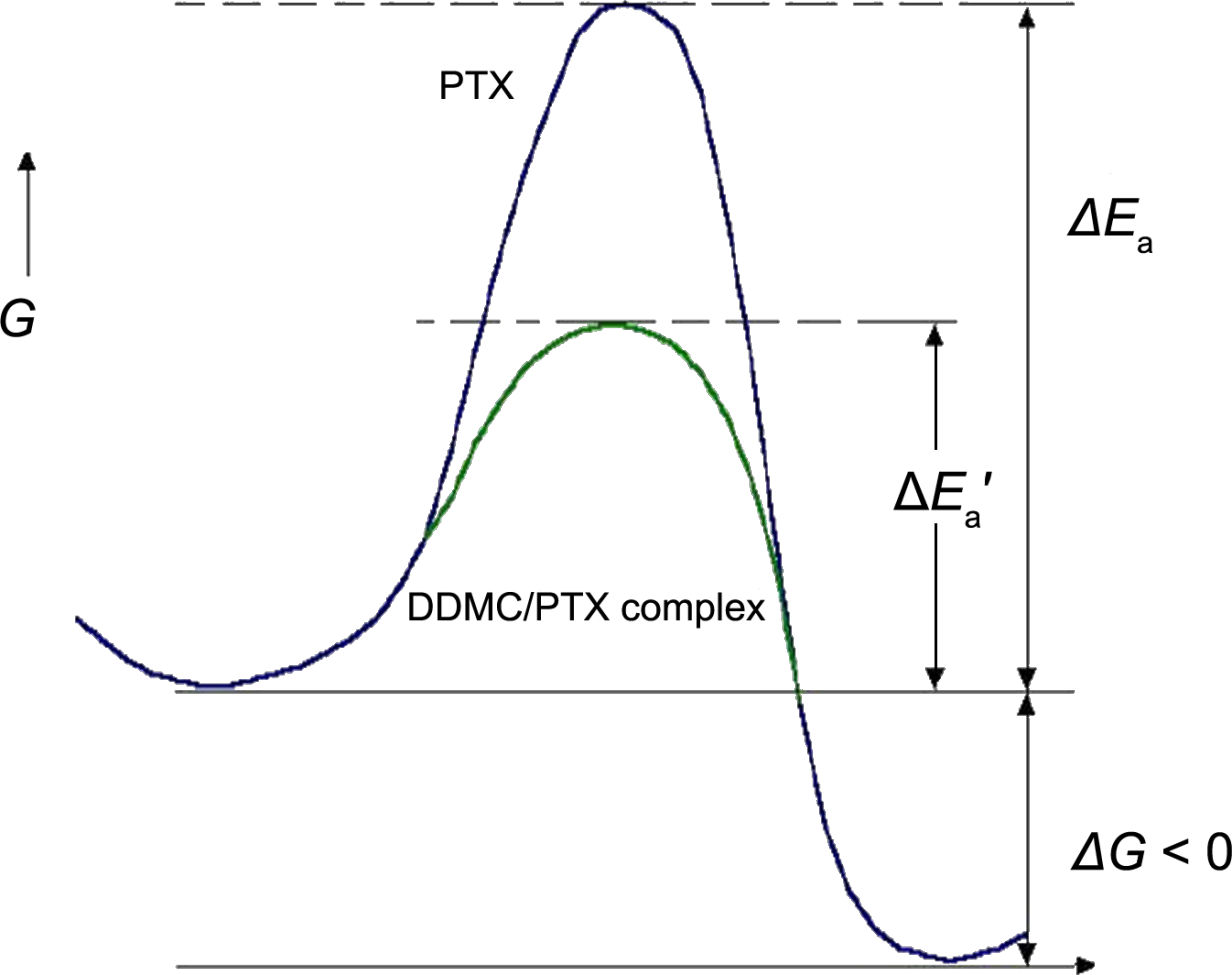
Potential energy curve of paclitaxel (large) and DDMC/PTX complex (small) in the tumor inhibition reaction. *E*_a_: activation energy of paclitaxel, *E*_a_′: activation energy of the DDMC/PTX complex. Reprinted from [24].

One hour after the administration of PTX, the RBM23 gene, which is a known target of PTX, acted as a hub and interacted with the TUBA4A gene encoded the tubulin α-4A chain. Two hours after administration, TXNIP became a hub gene, which was found to act as a key gene in the treatment of breast cancer that was not responsive to PTX. Four hours after administration, the mathematical model for the DNA microarray data revealed the activation of several genes downstream of EGR1 and TXNIP. Six hours after administration, CYR61, which is involved in resistance to PTX in breast cancer, became more active and continued to be influenced by EGR1. In this way, cancer cells exposed to anticancer drugs acquire resistance to the drug over time and show complex cellular behavior [37]. It can be understood that melanoma cells become resistant to PTX by changing their genetic control system. In this experiment, factor analysis was used to examine the effects of low PTX concentrations on the survival and gene expression programs of melanoma cells. However, at high concentrations, survival is inversely proportional to the PTX concentration. Truly, at high concentrations, PTX will be efficacious, and the promotion of tubulin polymerization by PTX, which is dependent on the PTX concentration, for a negative survival probability, will occur so that melanoma cells die. Therefore, the PTX concentration-dependent promotion of cell death (i.e., event B) becomes dominant and the efficacy of PTX becomes more apparent. This phenomenon means that the effect of PTX does not become remarkable, if, in other words, PTX does not overcome the potential MDR barrier from the resistance gene(s) of melanoma cells to low concentrations of PTX. The height of a potential barrier is called activation energy (as known in reaction kinetics), that is, a reaction molecule must exceed the large energy barrier of this process, and it turns out that a high concentration of PTX is required to achieve this, as shown in Figure 6.

By contrast, the responses of melanoma cells to the DDMC/PTX complex are much more specific without event A. Of note, low concentrations of the DDMC/PTX complex markedly inhibited the increase in the number of melanoma cells, with a linear negative correlation between PTX concentration and survival (Figure 5a and Figure 5b). This means melanoma cells have no resistance to the DDMC/PTX complex, as if this relationship is a series of intracellular enzyme reactions. In other words, the relationship between the cell death (Cd) rate (Cd/*dt*) and tubulin polymerization (p) rate (*dp*/*dt*) will assume the following equation, as modified from the Cheng and Prusoff equation [38–40]:

[1]
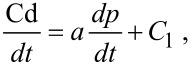


where, *a* is a constant value and *C*_1_ is a device constant (*a* > 0, *C*_1_ > 0).

#### Michaelis–Menten kinetics

The Michaelis–Menten equation assumes an enzyme reaction model with a ratio of 1:1 for a substrate and enzyme, and an S-shaped curve is derived for an allosteric environment, as follows [41]:

[2]
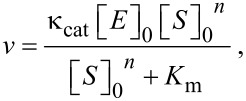


where *K*_m_ is the Michaelis constant, [*S*]_0_ is the initial tubulin concentration, and *n* is the Hill coefficient. In this case, because n ≥ 1, mutual interactions between numerous points occur, which fit an S-shaped curve.

Then, when Equation 2 is rewritten:

[3]
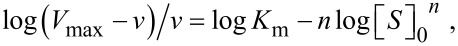


where *V*_max_ is the maximum enzyme reaction rate. The plot of log (*V*_max_ − *v*)/*v* by log [*S*]_0_*^n^* must be a straight line. The stability of the enzyme–substrate complex is shown as 1/*K*_m_, which should be larger for DDMC/PTX than for PTX alone when *n* = 1, corresponding to [*S*]_0_*^n^* ≥ *K*_m_. Therefore, the following equation is obtained:

[4]
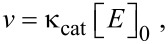


where [*E*]_0_ is the initial concentration of PTX, and the cell death rate in percent (Cd) is cited as the enzyme–substrate reaction rate *v* in Equation 1,

[5]
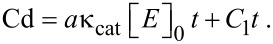


Here, C_1_ is a device constant. In Figure 5b, the incubation with MTT for 24 h yielded the following equation:

[6]
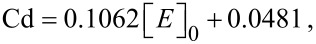


and for 48 h,

[7]
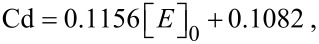


when *C*_1_*t* = 0.0481 at 24 h and 0.1082 at 48 h are plotted, it can almost be extrapolated to 0 at time *t* = 0 from Figure 5c. The DDMC/PTX supramolecular complex would adapt to the enzyme reaction model by an allosteric effect because it is fitted to the Michaelis–Menten formula under an allosteric environment here. As mentioned above, the mode of action of the DDMC/PTX complex may be considered as the same selective catalytic reaction as an enzyme reaction of the “lock and key” model [42], reflecting the supramolecular characteristic of allosteric promotion, as in Equation 2. In the system of the DDMC/PTX complex, it is thought that DDMC/PTX reacts to β-tubulin at the multi-enzyme reaction model ratio of 1:1. This shows the artificial enzyme and substrate specificities of the supramolecular characteristics on the inclusion complex that is composed of DDMC as a host and PTX as a guest. From its low concentration, this substrate specificity maintains a reaction with the tubulin protein, which is a target molecule of PTX, and various kinds of disturbances of the gene expression program for survival at low concentrations of PTX alone will be eliminated in the case of DDMC/PTX. Not being influenced by the above-mentioned resistance of melanoma cells to PTX, it may be able to react with tubulin protein, resulting in melanoma cells undergoing apoptosis. PTX can be conjugated to a variety of carriers, including polyglutamate [43] and albumin [44], encapsulated in cationic liposomes [45], or PEG-polyaspartate [46]. By using these carriers, PTX is thought to be transported into and released directly in cells, thus improving its efficacy. However, the DDMC/PTX complex will be not degraded in the cell, and its efficacy may be enhanced by remaining in its supramolecular form. It is thought that the tubulin polymerization reaction induced by the DDMC/PTX complex is promoted when the substrate is coordinated and polymerized at the active site (PTX).

In the case of transfection by using a non-viral vector, it is difficult to induce a nuclear shift, and the majority of gene expression from a DDMC/DNA complex may be outside of the nucleus after endocytosis, except during cell division. The introduction of a shift in location is considered an effect of the drug in the DDMC/PTX complex. ATP-binding cassette transporters translocate a wide variety of hydrophobic substrates with molecular weights of 300–2000 Da. With regard to the large molecular weight of DDMC of 1,000,000 Da, it would be impossible for ATP-binding cassette sub-family B member 5, also known as P-glycoprotein ABCB5, to react with the DDMC/PTX complex removing it from the cell [47]. If the complex decomposes within a cell, just like any other drug carrier, naturally, DDMC/PTX will be also eliminated from the cell by the ABCB5 transporter, etc., as a resistance mechanism of melanoma cells to PTX. However, the intact DDMC/PTX complex has been shown to avoid this process.

Now, it will be necessary to consider these phenomena thermodynamically as a cell death kinetic by an enzyme–substrate reaction. According to transition-state theory, if allosteric strain (distortion or strain) and entropy decrease, the “entropy trap” accompanying enzyme molecule recognition can be performed, the substrate will enter a transition state easily, and the activation energy will become lower. Conversely, in an enzyme–substrate reaction, since an adsorbed substrate is bound to an enzyme interface, its degree of freedom falls, and entropy generally will decrease. Therefore, in order for an enzyme–substrate reaction to advance spontaneously, it needs to be set to free energy change: Δ*G* = Δ*H* − *T*Δ*S* < 0, and enthalpy (Δ*H*) must also decrease greatly with the entropy (enthalpy–entropy compensation or isokinetic theory) [28]. In fact, the change of enthalpy, Δ*H*^‡^, will decrease greatly by reduction of the interface for substrate adsorption, which is known popularly as an “induced-fit model” [48]. The enthalpy change is almost equal to the activation energy (Δ*E*_a_) in Figure 6, and a DDMC/PTX complex promotes a tubulin polymerization reaction, thereby leading to the promotion of melanoma cell death, 
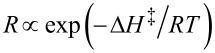
. Conversely, since a large enthalpy, that is, a large activation energy (Δ*E*_a_), is required for PTX alone, the only time the PTX concentration is increased more for the reason shown in Figure 5a, it will result in melanoma cell death.

#### Survival analysis on the anticancer activity of the DDMC/PTX supramolecular complex on melanoma cells in vivo

In vivo analysis of the anticancer activity and survival rates of the DDMC/PTX complex in B16F10 melanoma cells was carried by using 6-week-old C57BL/6 female mice with: PTX, DDMC/PTX4, DDMC/PTX5, and saline, following the regulations for animal experiments and related activities at Oita University (license no. N009001). To evaluate the antitumor effect of the DDMC/PTX complex, tumor-bearing mice were prepared by inoculating B16F10 melanoma cells subcutaneously in the back of C57BL/6 female mice (2.0 × 10^6^ cells/mouse). On average, 1885 mm^3^ of very severe tumor volume was observed. At 12 days after inoculation, PTX, DDMC/PTX4 (particle size 50 nm), DDMC/PTX5 (particle size 290 nm), and saline were administrated by intraperitoneal (I.P.) injection 3 times at a dose of 10 mg PTX/kg on days 12, 14, and 16.

#### MST 50% survival time

The effect of treatment was evaluated by using the median T/C ratio (MST 50% survival time; %T [treatment]/C [control]) of the relative survival time. The survival data are summarized in Table 2.

**Table 2:** The median survival time of DDMC, the median survival time (50%, MST) of the PTX, saline, DDMC/PTX4 (size: 50 nm) and DDMC/PTX5 (size: 290 nm) groups. MST is shown using T/C (MST: T; treated group/C; control group).

	saline (control)	PTX	MC/PTX4	DDMC/PTX5

time [h]	120	176	352	292
T/C	1.0	1.46	2.93	2.43

The median survival times (50%, MST) of the saline, PTX, DDMC/PTX4, and DDMC/PTX5 groups were 120 hours (T/C, 1.0), 176 hours (T/C, 1.46), 352 hours (T/C, 2.93), and 292 hours (T/C, 2.43), respectively. The supramolecular DDMC/PTX complex showed twice the effectiveness of PTX alone (*p* < 0.036). At the same time, 24 h after I.P. injection, the tumors of the DDMC/PTX4 and DDMC/PTX5 groups changed from a circle form to an ellipse form; however, one of the tumors in the PTX or saline group was still in a circle form. This tumor strain means that the DDMC/PTX complex inhibits cancer growth by promoting α,β-tubulin polymerization to induce strong stress to the cytoskeleton or an anti-angiogenic reaction. From this, the DDMC/PTX complex can induce a rapid and remarkable response. The difference between the PTX and DDMC/PTX groups was remarkable, and the validity of using the DDMC/PTX complex in the treatment of severe metastatic conditions was confirmed.

#### Tumor growth inhibitory activity

**Figure 7:**
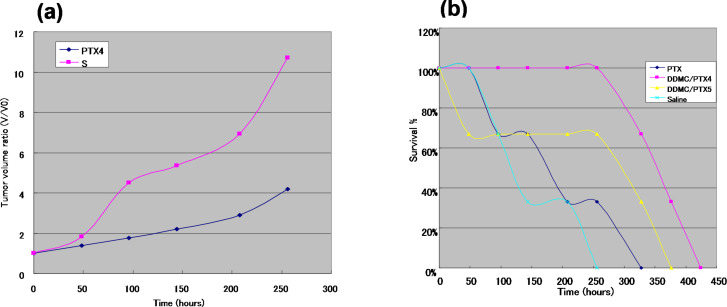
In vivo analysis of the anticancer activity and survival rates of the DDMC/PTX complex in B16F10 melanoma cells. (a) The increase rate (*V*/*V*_0_) of mean tumor volumes in the saline and DDMC/PTX4 (particle size: 50 nm) (*p* < 0.09). (b) The relationships between survival rate and time (hours) with the PTX, saline, DDMC/PTX4 (particle size: 50 nm) and DDMC/PTX5 (particle size: 290 nm) groups. (b): Reprinted from [62].

Tumor growth inhibitory activity was evaluated by using B16F10 melanoma cells in xenograft tumor-bearing C57BL/6 mice. As shown in Table 3, the tumors grew rapidly in size when the mice were treated with saline or PTX. However, treatment with the DDMC/PTX complex inhibited cancer growth more than saline and PTX, and showed remarkable cancer growth inhibition after 48 h from I.P. injection. The increase rates of mean tumor volume in the PTX, saline, DDMC/PTX4, and DDMC/PTX5 groups were 1.85, 1.84, 1.39, and 1.53, respectively. Although PTX alone was ineffective, tumor volume for DDMC/PTX4 treatment fell to 46% and 63% for DDMC/PTX5 treatment compared to the control. The relation between tumor volume ratio (*V*/*V*_0_) and time with control and PTX4 are shown in Figure 7a. However, while the control exhibits a malignant tumor which grows uncontrollably, the tumor growth in DDMC/PTX4 is controlled up to a degree (*p* < 0.09).

**Table 3:** Cancer growth (*V*/*V*_0_) after 48 h from 1st I.P. injection.

saline	PTX	DDMC/PTX4	DDMC/PTX5

1.84	1.85	1.39	1.53

#### Survival curves of mice

As shown in Figure 7b, very interestingly, the mice treated with saline or PTX showed a more gradual decrease in survival from the early time points of the experiment. Conversely, there appeared to be no change in survival for longer than 256 h in the DDMC/PTX4- and DDMC/PTX5-treated mice. Different chemo-effects on melanoma cells between the PTX- and DDMC/PTX-treated groups can be deduced from this result. It is thought that the survival of the mice was maintained by the probability of the suppression of metastatic tumor growth or the metastatic control of the B16F10 melanoma cells, which are a well-known model of pulmonary metastasis. It is possible that the DDMC/PTX complex, which has the ability for angiogenesis prevention, growth inhibition, and suppression of fatal metastasis on B16F10 melanoma cells, is effective by these mechanisms. The efficacy of DDMC/PTX4 (MST: T/C, 2.93) treatment to inhibit melanoma tumor growth was also superior to DDMC/PTX5 (MST: T/C, 2.43) treatment, owing to its small particle size of 50 nm (EPR effect) (*p* < 0.0033). The in vivo drug efficacy of DDMC/PTX on cancer cells should depend on its EPR effect, avoidance of the RES, and its artificial enzymatic function.

#### Case study of murine melanoma

**Figure 8:**
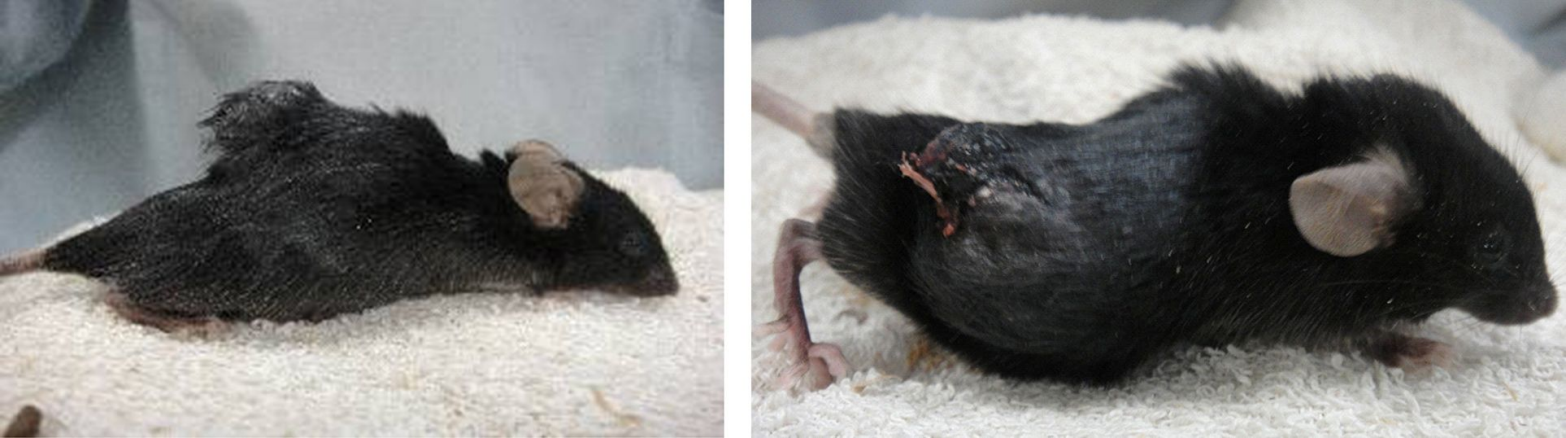
Two mice of both PTX (left) and DDMC/PTX4 (right) groups after 208 hours from I.P. In the mice of DDMC/PTX4 group necrosis was induced by TNF-α. The increase of TNF-α in an individual has been checked by the phoresis results after RT-PCR. Reprinted from [62].

Figure 8 shows two mice from the PTX and DDMC/PTX4 groups at 208 hours after I.P. injection. A mouse in the DDMC/PTX4 group was almost cured after small dermatorrhagia owing to the anti-angiogenic effect of the treatment. The DDMC/PTX complex reacts better to melanoma cells at the point of metastasis. This seemed to be reflected by the hemorrhagic necrosis of the tumor, which was considered to be caused by the discharge of the tumor necrosis factor alpha (TNF-α) cytokine by M1 macrophages. Later, the increase in TNF-α levels and the decrease of eNOS in individual mice was checked using phoresis and the RT-PCR findings of the mouse in the DDMC/PTX group. The down-regulation of eNOS (an angiogenesis regulator) protein should be predominantly carried out by using RNA interference induced by miR-222 following “Argonaute/RISK” of high polymer DDMC/PTX [49].

It is known that the TLR3/TICAM-1 pathway of M1 macrophages in a tumor is the key to TNF-α production [50]. PTX reportedly affects the native or acquired immune response, thereby inducing M1 macrophages, which secrete NO and proinflammatory cytokines such as IL-1β, GM-CSF, and TNF-α [51]. Although it has been thought that M1 and M2 macrophages are important factors for cancer recovery, it was noted recently that the grade of malignancy was high when many M2 macrophages infiltrate a tumor [52]. This is why M2 macrophages have an important role in the angiogenesis or lymphangiogenesis of malignant cancer cells.

Shime et al. [50] reported that malignant cancer cells act on the adjuvants of natural immunity depending on the presence of double-stranded RNA, and M2 macrophages change into type M1 macrophages during cancer to attack malignant cells. Horlad et al. [53] also showed that triterpenoid compounds such as corosolic acid isolated from apples, which activates the glucose transporter and suppresses the rise of blood glucose levels, inhibit the signal transduction factors related to the differentiation to M2 macrophages, namely, nuclear factor-kappa B (NF-κB) and signal transducer and activator of transcription 3 (Stat3), and the tumor is targeted with an aim to recovery. Stat3 is activated by tumor-derived factors and macrophages differentiate into the M2 lineage [54]. Thus, the importance of M2 macrophages on the angiogenesis or lymphangiogenesis of metastatic cancer is beginning to be recognized strongly.

Regarding the differentiation of M2 macrophages, the DDMC/PTX complex does not act it to invoke an immune response having stealth nature in the original as having a hydrophilic–hydrophobic microseparated-domain with an amphiphilic domain (DEAE-dextran) and a hydrophobic domain (PMMA) [21]. Thus, it is difficult for this complex to be decomposed by foreign substance recognition. As the drug interaction of the DDMC/PTX complex is in accordance with a Michaelis–Menten-style multi-enzyme reaction model of a 1:1 ratio of a substrate and enzyme, a very small amount of proinflammatory cytokines must be released, leading to M2 macrophage differentiation. It seems that it is not very rare to receive useless inflammatory signals in an enzyme reaction model. From another point of view, it is imagined that the production of M2 macrophages by the differentiation of M1 macrophages through the jumonji domain-containing histone demethylase (Jmjd3) pathway does not occur readily following DDMC treatment as it contains α-1,6 glycoside linkages, differing from chitosans or celluloses (β-1,4), which are constituent materials of parasites, insects, and filamentous bacteria [54]. As a result, the medicinal action of the DDMC/PTX complex will suppress the tumor-associated action of M2 macrophages, and will go in the direction that controls the multiplication of cancer cells [51].

### Artificial enzyme

#### Supramolecular allosteric effect

PTX is isolated from the bark of the yew tree, and it combines with β-tubulin to promote polymerization by stopping the treadmill of tubulin protomers. MDR of cancer cells to PTX is based on the presence of, for example, mutant tubulin, or by a glutathione-mediated reaction.

#### Enzyme–substrate interactions

**Figure 9:**
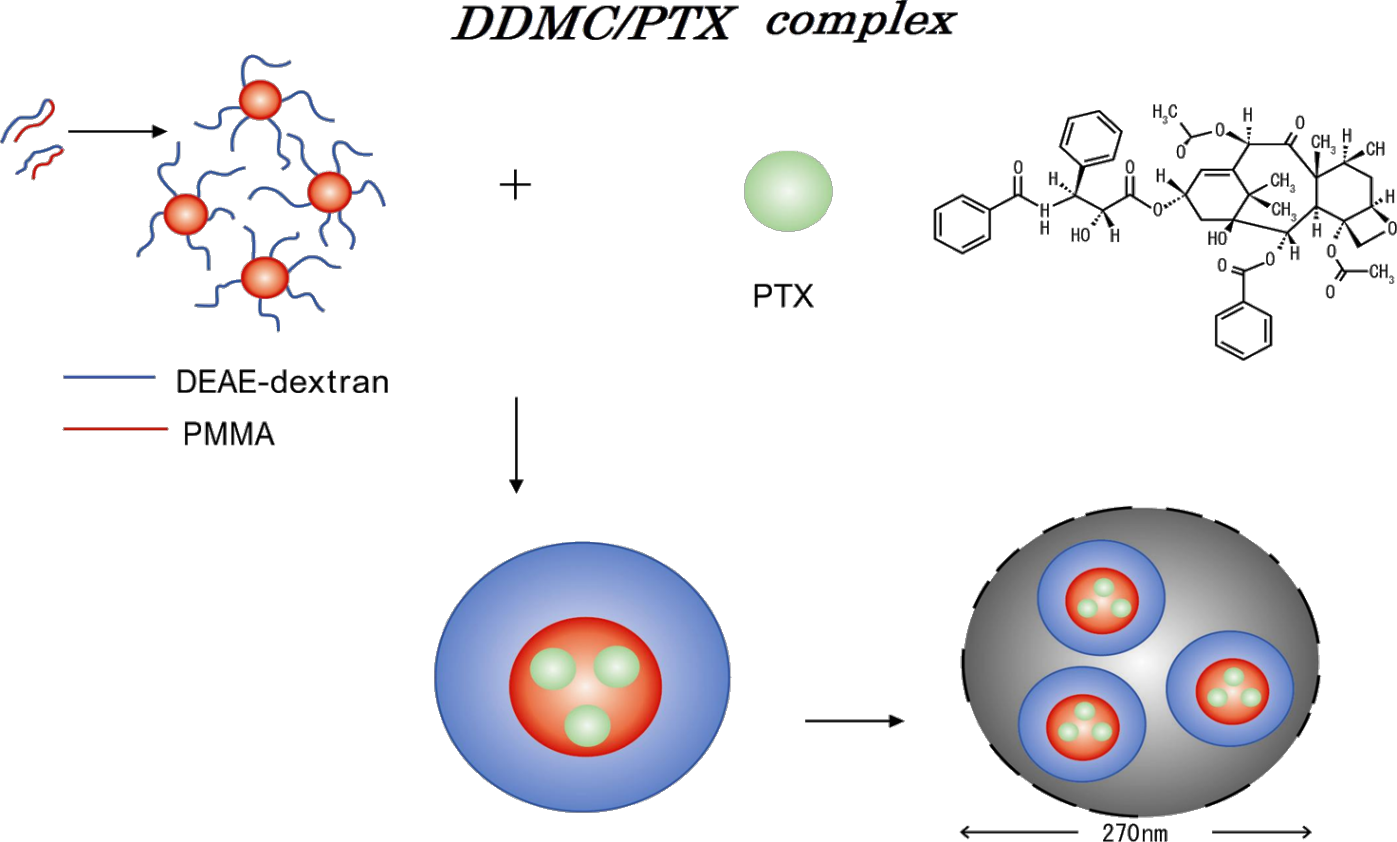
α,β-tubulin dimer orientates with the active site (i.e., paclitaxel) of the DDMC/PTC complex. The DDMC/PTX complex (ca. 270 nm) will consists of more than 8.1·10^3^ DDMC molecules and 6.7·10^6^ paclitaxel molecules. Reprinted from [24].

**Figure 10:**
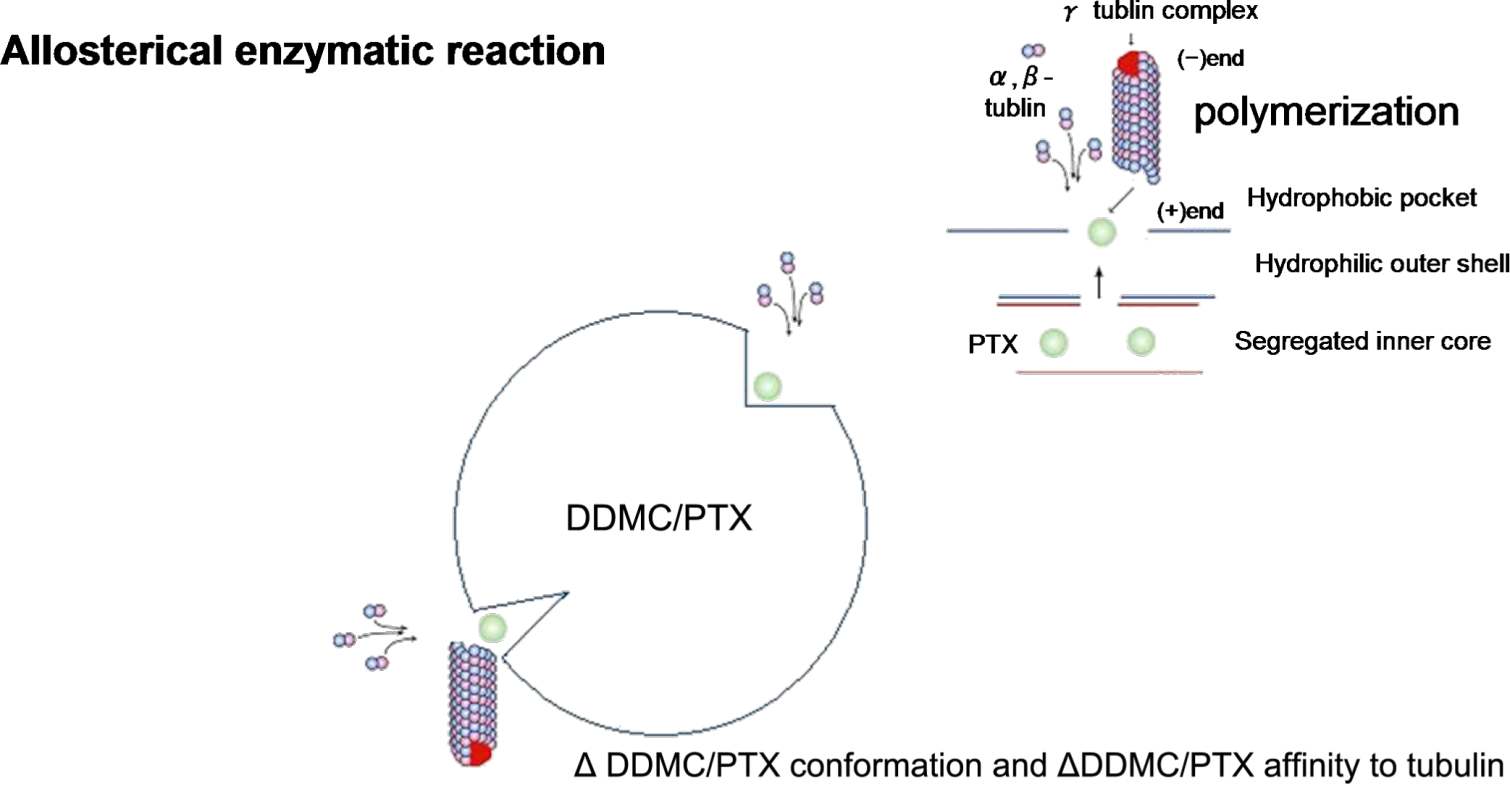
Schematic representation of α,β-tubulin dimer polymerization showing the allosteric relationship between the DDMC/PTX complex and α,β-tubulin. Simplified structures are shown. Reprinted from [62].

Thus, the allosteric properties of these supramolecular assemblies resemble those of enzyme–substrate interactions. For hemoglobin, oxygen acts as an effector and as the substrate. Binding of the molecule to the allosteric binding site on one subunit enhances the affinity of the other binding sites by inducing structural changes, as explained by a sigmoid curve. The results indicate that these enzymatic reactions promote allosterically the polymerization of tubulin in cells treated with the DDMC/PTX complex, which has slide-ring elasticity [29,32]. In the DDMC/PTX complex, hydrophobic interactions between the polymer and substrate, with PTX located in so-called “hydrophobic pocket,” allow PTX to react selectively with tubulin as indicated in Figure 9 and Figure 10. This effect is not apparent with PTX alone, and will be less susceptible to interference by other signals from cancer cells. Allosteric enzymatic reactions in the α,β-tubulin dimer must be effective on the conformational changes that occur in the presence of the drug [55].

The results also provide evidence that the DDMC/PTX complex causes a supramolecular reaction involving allosteric promotion as in Equation 2. The Hill coefficient included in Equation 2 supports the likelihood of allosteric cooperation as it represents the strength of cooperative molecular joints. These may depend on the supramolecular facility (entropy elasticity) of clathrate compounds, such as PTX as the guest, which is complexed with DDMC as the host. Therefore, the DDMC/PTX complex shows marked substrate specificity as an artificial enzyme. The substrate specificity of the DDMC/PTX complex promotes enzymatically a reaction between PTX and β-tubulin and avoids potential interference from any changes in gene expression that may affect the survival response to PTX, even at low concentrations. Thus, the DDMC/PTX complex may not be degraded in the cell, and its efficacy may be enhanced by remaining in a supramolecular form. The DDMC/PTX complex is formed by self-assembly and aggregation, forming particles of approximately 50–270 nm in size. Electrostatic and hydrophobic interactions between the cationic DDMC/PTX complex and its substrate, the α,β-tubulin dimer, orientate β-tubulin with the hydrophobic pocket of the DDMC/PTX complex, which promotes the polymerization of α,β-tubulin dimers through the activity of PTX by their entropy elasticity, which is consistent with allosteric Michaelis–Menten kinetics. The stability of the enzyme (DDMC/PTX complex) substrate (α,β-tubulin dimer) complex (Michaelis complex) can be represented as 1/*K*_m_, which is probably small [42] for an allosteric reaction; it does not cause dynamic instability in the tubulin dimers to stop the treadmill of tubulin protomers.

#### Tubulin polymerization

This α,β-tubulin dimer and DDMC/PTX complex also contains Mg^2+^ and GTP on the leading edge, which may also be involved in tubulin polymerization, as follows:

[8]
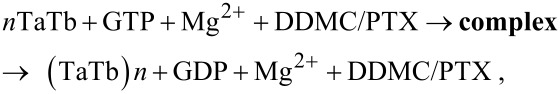


where TaTb is the α,β-tubulin dimer, and DDMC/PTX acts as a biocatalyst.

The α-tubulin side is a negative edge, and the β-tubulin side is a positive leading edge. Most microtubules are composed of α,β-tubulin protomers bound to a molecule of GDP. During polymerization, both the α- and β-subunits of the tubulin dimer are bound to a molecule of GTP. Many microtubules place a negative edge in the microtubule organizing center (MTOC), the centrosome in an animal cell, to extend the (+) growth edge to each place of the cell. Of course, other microtubules that form outside of the centrosome are identified and transported to the MTOC [56]. In one situation, the tubulin-binding agent is a microtubule-stabilizing agent.

Cell division is usually initiated when polymerization ceases to form a sufficiently long hollow tube to provide support for myosin and actin located on the cell surface by gathering its 13 fibers. The DDMC/PTX complex stabilizes the α,β-tubulin dimer, which is therefore unable to stop polymerization, promoting cell apoptosis following the formation of numerous poor-quality short hollow tubes [55].

#### Enzymatic reactions of the DDMC/PTX complex

Figure 9 and Figure 10 show the diffusion of PTX to the outer surface, the coordination of α,β-tubulin dimer on the DDMC/PTX complex, and the subsequent growth of the α,β-tubulin dimer on DDMC/PTX. This reaction may be accelerated by the orientation of the α,β-tubulin dimer to PTX within the “hydrophobic pocket”" of the DDMC/PTX complex. Furthermore, the growth reaction of the α,β-tubulin dimer is accelerated and apoptosis is induced. The feature of this coordinated polymerization as it makes a series of enzyme reactions is that both ends of a tubulin dimer and a tubulin growth chain are configured to progress on a supramolecular assembly. This will generate large substrate selectivity of tubulin and stop MDR from emerging. As revealed with PTX, a common antimicrotubule drug, MDR appears unable to form adequate medicine–substrate conjugation according to β-tubulin subtype (class I, II, III, IVa, IVb, and VI) [57–60]. It has been reported that Taxol can act only on normal tubulin polymers, it cannot act on the unstable β-tubulin class III or IV subtypes, and MDR will occur [59,61]. For PTX, β-tubulin already loses its characteristic as a substrate by the change of its conformation to a subtype that cannot participate in the polymerization reaction of α,β-tubulin. So, for the β-tubulin subtype, Taxol loses its medicinal effect, which prevents cell division [59]. However, the DDMC/PTX complex has a substrate specificity that cancels the conformation change for its comparatively stable 1/*K*_m_, owing to its large *K*_m_ from the sigmoid curve for an allosteric reaction, with a β-tubulin subtype by the allosteric effect, when it has conformational flexibility on its supramolecular structure (entropy elasticity), similar to an allosteric enzyme. From this supramolecular DDMC/PTX complex of an artificial enzyme with allosteric action and substrate selectivity, as shown in Figure 10, it maintains its medicinal effect, which corresponds to a conformation change of a β-tubulin subtype and reacts quickly to it. Therefore, it will not lose its efficacy as a drug [24,62–63].

## Conclusion

Above all, with respect to MDR of cancer cells by the existence of β-tubulin mutations or a glutathione-mediated reaction, the supramolecular DDMC/PTX complex can oppose the development of MDR through effective tubulin substrate selectivity and an allosteric method of action. The DDMC/PTX complex showed superior anticancer activity to PTX alone; it had a linear relationship between the dose and cell death rate determined using allosteric Michaelis–Menten kinetics applied to an enzymatic reaction. This shows that the complex promoted an allosteric supramolecular reaction to tubulin as an artificial enzyme. From our results, the DDMC/PTX complex should not be degraded significantly in cells and will achieve good efficacy as an intact supramolecular anticancer agent. Above all, the supramolecular complex by DDMC/PTX should play an important role as an amplifier of the PTX drug efficacy [64].

## Statement for studies with animals

The animal studies were carried out by the authors following the guidelines of Oita University for the care and use of laboratory animals.
